# Calcification

**Published:** 1890-08

**Authors:** Sidney Spokes


ARTICLE II.

### CALCIFICATION.

BY SIDNEY SPOKES, M.R.C.S., L.D.S.

When the Dental Student has made himself acquainted with the arrangement of the soft tissues which constitute the formative organs of the future tooth, he has next to consider the process by which these soft structures become changed into the hard organs known as teeth.

In addition to other observations bearing upon this subject, a paper on the "Secretion of Lime by Animals" has recently appeared in the Report issued from the Laboratory of the Royal College of Physicians of Edinburgh. Mr. Invine and Dr. Sims Woodhead point out that the carbonate of lime present in sea-water is only one tenth of the whole lime salts present, whereas in the coral, shells and calcareous plants formed from the sea by animal and vegetable life the whole line is practically in the form of carbonate. On the other hand, sulphate of lime is largely present in sea-water, and these observers thought that this salt might be assimilated and then elaborated as carbonate. They experimented upon laying hens, the only lime supplied to the birds being the pure hydrated sulphate. It was calculated that during six weeks 954 grains of carbonate were elaborated in the form of egg-shell from the sulphate of lime supplied. On examining the oviduct it was found to be secreting lime, the epithelial cells containing lime in minute granules which, incorporated with the organic matter, appear to form the egg-shell. "It is interesting to note in this connection that the epithelial cells are evidently in a state of great functional activity, as a consequence of which the nucleus is considerably obscured, and that along with the lime there is secreted some other substance (probably the organic material in which the lime is eventually embedded.)" Having pointed out how the sulphate of lime may be changed into the carbonate these observers again refer to the special function of the epithelial cells in secreting the lime brought to them by the blood and lymph. "This lime evidently accumulates in the epithelial cells and is then thrown out on the free surface, just as urate, oxalates of lime, soda, &c., are secreted by the renal epithelium." As a result of the tissue waste by the activity of the cells, carbonic acid is present to combine with the lime secreted, and an analogy is drawn to what occurs in the formation of bone where, it is said, the organic basis is laid down by cells which, at the same time, actually secrete the salts of lime derived from the blood and lymph. "The osteoblasts are the secreting cells." This statement may perplex some students who, perhaps, are taught that bone is formed by the ossification of the osteoblasts themselves; and perhaps some exception may be taken to the analogy being true. Another exception is pointed out by the authors—that in the formation of bone, where the amount of phosphoric acid salts in the blood and lymph is large and there is no special development of carbonic acid gas, the phosphates predominate. Whilst it is admitted that the above processes are "merely suggested," those interested in the subject will be glad to know that further investigations are promised; the great difficulty, it is said, being the ignorance at present existing as to the changes effected by protoplasm upon the constitution of inorganic salts. With regard to the conversion of lime salts, and secretion of carbonates, the authors say "that the process is the result of a combination of two forms of activity, the purely chemical and what we must call vital, must be taken for granted; and it is this vital part of the process which appears to eke out the possibilities of chemical reactions taking place." While it may be strictly true that secretion can only take place in a living medium, it may perhaps be asked whether it is possible to regard the conversion of soft organized material into a more or less hard mass as necessarily a vital process; for it has been done in the laboratory. Leaving on one side for the moment the consideration as to whether the cells concerned are themselves the receptacles for deposition of lime salts or whether they secrete something which afterwards becomes impregnated, the larger question presents itself—what is the relation, chemical or vital, between the inorganic salt and the organized material? In connection with this, every well regulated student bears in mind the names of Rainie, Ord, and Harting, and it may be useful to present, although in a necessarily condensed form, some account of their observations, if only to save reference to distinct papers.

Rainie, in 1858, published a book "On the mode of formation of Shells of Animals, of Bone, and of several other structures by a process of Molecular Coalescence, demonstrable by certain artificially formed products." Amongst other things he claimed to have discovered a process by which carbonate of lime can be made to assume a globular form, and he explained "molecular coalescence," the process by which that globular form is produced. He also came to the conclusion that the rounded forms of organized animal bodies depended upon physical and not on vital agencies. In a broad-mouthed bottle a solution of gum arabic saturated with carbonate of potash was allowed to stand until the carbonate of lime set free from the gum and the triple phosphate set free by the alkali were settled at the bottom. Two clean microscope glass slides, inclined against each other, were inserted, and the bottle filled up carefully with a filtered solution of gum arabic in common water. It was then covered up and kept perfectly still for three weeks or a month. The soluble salts of lime to be decomposed by the carbonate of potash are contained in the gum (in combination with the malic acid) and also in the common water; triple phosphate is also contained in the gum and set free by the alkali. The deposit formed upon the slides in the gum is of a globular form instead of the crystalline found in ordinary solutions.

Dr. Ord, 1870, found by experiment that where uric acid was deposited in the presence of albumen it took the form of either small crystals with rounded angles, or of dumb-bells, or of sub-spherical bodies or even spheres. Glass tubes with a plug of isinglass jelly at the lower end were placed in a weak solution of chloride of calcium and then filled up with a slightly alkaline solution of potassium oxalate. After three months the plug was opaque with earthy deposit and stratified, forming a layer of greatest density near the calcium solution. The forms varied on the oxalate side from those on the calcium, and a series of gradations led from one to the other; the coalescence forms were chracteristic of the calcic end, the crystalline of the oxalic end of the plug. Very many other experiments were made. When gelatin was used and magnetized, the general result was an extraordinary increase in the size of all the forms, crystalline and non-crystalline, but no new forms nor a greater tendency to sphericity. The effects of light and heat were tried; no decisive results were gained in the former, but in the latter a kitchen specimen gave coalescence forms three or four times as numerous as the crystalline, whilst in a garden specimen this condition was more than reversed. Albumen was coagulated in tubes at a temperature not exceeding 200°, and oxalate of calcium deposited in these plugs at a temperature of form 50° to 60° took almost entirely the coalescence forms. Albumen was evidently much more active than gelatin in controlling crystallizing force. When phosphates and carbonate were arranged in proper proportions, it was found that the phosphate of lime was evenly distributed through the albumen in definite strata, not forming crystals or spheres, but cementing the albumen to great hardness, particularly at the line of greatest density. Carbonate of lime always formed spheres at the higher temperatures, but at lower temperatures showed attempts at crystallization by the formation of spines sticking out from the the spheres. In the bone-salts experiments the carbonate of lime was subdued, so to speak, by the phosphate, and an even sub-crystalline but continuous deposit was produced. The use of a phosphate as a cement and manipulator of the less tractable carbonate, is well indicated in these experiments; the strength of the carbonate seems necessary in all hard tissues that have to be tough. But the carbonate alone does not seem fitted to form tissue, tissue destined to be the seat of active interstitial change, therefore in the bird's high temperature and great vital activity we see associated a great predominance of phosphate. In the tortoise, with its low temperature and sluggish processes, a great decrease of phosphate and increase of carbonate; and in the shells of Invertebrata, where interstitial change does not prevail, the carbonate alone or with but little phosphate suffices. In cases where this does not seem to hold good the explanation may be found in the nature of the animal matter with which the salts are associated.

Professor Harting published "Researches in Synthetical Morphology on the Artificial Production of Some Organic Calcareous Formations." He caused calcium carbonate and phosphate to combine, *in the nascent state*, with organic substances by using salts which by their double decomposition produce insoluble salts of calcium. After several weeks a considerable number of forms are developed which are mostly found in nature. The most frequently occurring form affected by calcium carbonate, in combination with albumen, gelatin, or the other organic substances used, he called *Calcospherites*. When these are formed in the midst of the liquid and the surrounding parts are perfectly tranquil, they are quite spherical; they become larger in proportion as their formation takes place with greater tranquility and slowness. They often contain a nucleus, and all those which attain a certain size are seen to be formed of concentric layers and very fine radiating fibres. Similar calcospherites, of spheroidal shape, are met with in nature in the form of different concretions in the bile, urine, and the saliva of certain animals; pearls are calcospherites, which in the course of time have attained remarkable dimensions. When the fluid is not kept quiet other forms occur, modifications of the calcospherite; one very remarkable is called *conostat*, characterized by a cup-shaped enlargement which becomes filled with air, and floats. The most remarkable of all the conditions, which assist in determining the shape of the calcospherites, consists in the fact of their mutual adhesion when they are developed in the neighborhood of one another. Calcospherites consist of a combination of calcium carbonate with organic matter, which is the sole residue when the calcareous salt is removed by treatment with an acid. If the development has taken place in albumen, or a liquid containing albumen, the fundamental substance is no longer albumen. The albumen is transformed into a substance, the chemical relations of which are those of conchyoline, and resemble those of chitin. This Harting calls *calcoglobulin.* It is not necessary however to cause albumen to combine with calcium carbonate and then to decompose the resulting compound in order to obtain calcoglobulin. It can be obtained by placing a fragment of chloride of calcium in albumen. After some days the albumen dissolves the calcareous salt and is transformed into calcoglobulin, which presents also, in part, a fibrillar structure, and, having been washed, gives all the reactions of that substance.

If calcium phosphate is liberated by the double decomposition of calcium chloride and a neutral phosphate in albumen or gelatin no combination takes place. But if calcium carbonate is present there is a combination of the organic matter with the two calcareous salts.

From the above quotations it will be seen that it is not difficult for any student to make calcospherites for examination, and thus prepare the way for understanding better the relationship of the cell and the lime salt in the living body.

With regard to the question as to the conversion of the odontoblast in the formation of dentine, there is a passage in the paper communicated by Mr. C. S. Tomes to the Royal Society, "On the structure and development of Vascular Dentine," which seems to have escaped the textbooks. In describing the capillaries clothed with odontoblasts, it is pointed out that when the latter calcify the capillary becomes solidly embedded in dentine. "And this is a strong argument in favor of what is known as the "conversion theory," of the development of dentine; supposing these odontoblasts to be calcified and themselves converted into dentine, there is no difficulty in seeing how the capillary comes to be enclosed in a tube of dentine, having the same calibre as itself. But if the odontoblast cells secrete

2

the dentine, as maintained by Hertz and others, how is the process to be completed when there is no longer room for an entire odontoblast or the half of an odontoblast between the rigid wall of already formed dentine and the capillary? One can hardly conceive a secreting cell going on shedding out from its end its secretion when it has been reduced to, say, one tenth of its length; and unless one is prepared to accept such a conception, this observation of the structure of a Hake's tooth-pulp becomes fatal to any "secretion" hypothesis of the formation of dentine."—*London Dental Record.*

